# Prevalence and outcome of anemia among children hospitalized for pneumonia and their risk of mortality in a developing country

**DOI:** 10.1038/s41598-022-14818-2

**Published:** 2022-06-24

**Authors:** Mohammod Jobayer Chisti, Chowdhury Ali Kawser, Abu Sayem Mirza Md Hasibur Rahman, Abu Sadat Mohammad Sayeem Bin Shahid, Farzana Afroze, K. M. Shahunja, Lubaba Shahrin, Monira Sarmin, Sharika Nuzhat, Ahmed Ehsanur Rahman, Tahmina Alam, Irin Parvin, M. S. T. Mahmuda Ackhter, Gazi Md. Salahuddin Mamun, Shamsun Nahar Shaima, Abu Syed Golam Faruque, Tahmeed Ahmed

**Affiliations:** 1 International Centre for Diarrhoeal Disease Research, Bangladesh (icddr,b), Dhaka, Bangladesh; 2 Institute for Social Science Research, University of Queensland, Brisbane, Australia

**Keywords:** Clinical microbiology, Gastrointestinal diseases, Infection, Comorbidities, Digestive signs and symptoms, Fever, Hypoxia, Neurological manifestations, Respiratory signs and symptoms, Gastrointestinal diseases, Neurological disorders, Nutrition disorders, Respiratory tract diseases

## Abstract

Data are limited on the prevalence and outcome of anemia and its risk on mortality among children under five years of age hospitalized for pneumonia/severe pneumonia. Thus, we conducted a secondary analysis of data extracted from Dhaka Hospital of International Centre for Diarrhoeal Disease Research, Bangladesh to address the evidence gap. Among 3468 children fulfilling the study criteria,1712 (49.4%) had anemia. If children aged ≤ 1.0, > 1.0 to 2.0, > 2.0 to < 6.0, and ≥ 6.0 to 59 months had blood hemoglobin (Hb) value of ≤ 10.7 g/dL, ≤ 9.4 g/dL, ≤ 9.5 g/dL, and ≤ 11 g/dl respectively; we considered them anemic. The trend of prevalence of anemia was found to be inversely related to increasing age (Chi-square for linear trend analysis was done to understand the relation of anemia with increasing age, which was = 6.96; p = 0.008). During hospitalization anemic children more often developed respiratory failure (7.2% vs. 4.4%, p < 0.001) and fatal outcome (7.1.0% vs. 4.2%, p < 0.001) than the children who did not have anemia. After adjusting for potential confounders, such as female sex, lack of immunization, abnormal mental status, severe acute malnutrition, dehydration, hypoxemia, severe sepsis, and bacteremia using multivariable logistic regression analysis, anemia was found to be independently associated with fatal outcome (OR = 1.88, 95% CI 1.23–2.89, p = 0.004). Thus, future interventional studies on the early management of anemia may be warranted to understand whether the intervention reduces the morbidity and deaths in such children.

## Introduction

Pneumonia has been the major infectious cause of global under-five deaths since the late eighties^1^. Each year, it accounts for an estimated 0.8 million under-five deaths globally^2^. More than 95% of these deaths occur in lower- and middle-income countries^3^. Pneumonia causes estimated 12,000 under-five deaths yearly in Bangladesh, making it the largest killer among this age group^2^. Globally, childhood pneumonia related mortality reduction was 54% from 2000 to 0218^4^. Moreover, it is also important to note that after 2000, annual pneumonia related mortality declined significantly in Bangladesh, which potentially helped Bangladesh in attaining Millennium Development Goal. Data from the resource limited settings disclosed several risk factors for deaths from childhood pneumonia. Based on that, World Health Organization (WHO) emanated an endorsement that includes incorporating vaccines, appropriate antibiotics, oxygen therapy and other routine clinical care^5^. That helped lessen global pneumonia-related deaths between 2000 and 2018 by 54% among children under-five years of age^4^. We need to further bolster the reduction of pneumonia related mortality to attain the sustainable development goal. On the other hand, the prevalence of anemia was high, ranging from 10.2 to 56.6%, in children under five years of age, and the differences in prevalence occurred mainly due to the association of anemia with different co-morbidities among hospitalized children^6–8^. A number of co-morbidities were identified that affect the severity and outcome of anemic children that includes malnutrition^6^, pneumonia^7,9^, low birth weight^7^, poor socio-economic condition^8^, and intestinal parasitic infections^8^.

In a recent study^9^ presence of anemia in children hospitalized for pneumonia substantially increases the risk of severity of the disease and consequently was found to be one of the independent risk factors for deaths during hospitalization. If we are able to understand the role of anemia in pneumonia related mortality, especially in Bangladesh, that may help design potential future interventions and further help to achieve the sustainable development goal.. Thus, there is further need to have more conclusive evidence on the burden and role of anemia with other co-morbid conditions, including pneumonia and their potential outcome during hospitalization in Bangladesh. To further advocate the option of potential management of anemia in the early stage, we decided to investigate the prevalence of anemia and whether co-morbid conditions of anemia had any impact on the fatal outcome among children under five years of age hospitalized for pneumonia/severe pneumonia in Bangladesh.

## Materials and methods

### Study site

The study was performed in Dhaka Hospital of International Centre for Diarrhoeal Disease Research, Bangladesh (icddr,b), Dhaka, Bangladesh. The description of Dhaka Hospital has been provided somewhere else^10^.

### Study population

Children aged 0–59 months with pneumonia/severe pneumonia, admitted to Dhaka Hospital of icddr,b between August’2013 and December’2017, whose hemoglobin (Hb) level was measured, constituted the study population.

### Design

The data were extracted from electronic database of Dhaka Hospital of icddr,b. We stratified the study children into an anemic and non-anemic group. Anemia was defined if blood Hb values were ≤ 10.7 g/dL, ≤ 9.4 g/dL, ≤ 9.5 g/dL, and ≤ 11 g/dl among children aged ≤ 1.0, > 1.0 to 2.0, > 2.0 to < 6.0, and ≥ 6.0 to 59 months of age respectively^11,12^. The attending physician decided the selection for Hb investigation and was done in our icddr,b laboratory. An initial baseline comparison was made between the children who had anemia and those who did not have anemia. The final comparison was made between the study children who had a fatal outcome and those who survived during hospitalization.

### Patient management

Study children received management according to standard treatment following hospital guideline that has been described elsewhere^13^.

### Measurements

We have used pre-tested case report forms for initial acquisition of study relevant data from the electronic database before incorporating them in to a personal computer. We have evaluated baseline characteristics (Table 1) and outcome (Table 2) of children hospitalized for pneumonia/severe pneumonia with anemia compared to those without anemia. We further evaluated the characteristics (including anemia) of children who survived or expired after being hospitalized with pneumonia/severe pneumonia (Table 3).

**Table 1:** Baseline characteristics of under-five children with anemia hospitalized for pneumonia/severe pneumonia.

Baseline characteristics	All patients	Anemia		RR (95% CI)	p value
		Yes (n = 1712)	No (n = 1756)		
Age (months—median, IQR)	3468	9.05 (6.33, 12.69)	6.00 (3.89, 10.52)	–	< 0.001
Male (N)	2263	1121 (65.5%)	1142 (65.0%)	1.01 (0.96–1.06)	0.783
Lack of immunization (N)	480	235 (17.7%)	245 (18.2%)	0.98 (0.83–1.15)	0.760
Duration of fever (days—median, IQR)	2081	3.0 (2.0, 5.0)	3.0 (2.0, 5.0)	–	0.006
Duration of cough (days—median, IQR)	2342	4.0 (3.0, 7.0)	5.0 (3.0, 7.0)	–	0.016
Duration of respiratory difficulties (hours—median, IQR)	897	48.0 (24.0, 96.0)	48.0 (24.0, 96.0)	–	0.035
Severe acute malnutrition (N)	1522	781 (45.6%)	741 (42.2%)	1.08 (1.01–1.17)	0.042
Hypoxemia (N)	1083	585 (34.2%)	498 (28.4%)	1.20 (1.09–1.33)	< 0.001
Invasive diarrhea (N)	138	77 (4.5%)	61 (3.5%)	1.29 (0.93–1.80)	0.123
Dehydration (N)	837	371 (21.7%)	466 (26.5%)	0.82 (0.72–0.92)	< 0.001
Severe sepsis (N)	370	203 (11.9%)	167 (9.5%)	1.25 (1.03–1.51)	0.025
Bacteremia (N)	274	164 (16.3%)	110 (13.6%)	1.20 (0.96–1.49)	0.116

*N* total number of participants, *RR* relative risk, *CI* confidence interval, *IQR* inter-quartile range, () %.

**Table 2:** Outcome of under-five children with anemia hospitalized for pneumonia/severe pneumonia.

Outcome characteristics	All patients	Anemia		RR (95% CI)	p value
		Yes (n = 1712)	No (n = 1756)		
Respiratory failure	202	124 (7.2%)	78 (4.4%)	1.63 (1.24–2.15)	< 0.001
Duration of hospital stay (days—median, IQR)	3468	6.0 (4.0, 9.0)	6.0 (4.0, 9.0)	–	0.457
Death	194	121 (7.1%)	73 (4.2%)	1.70 (1.28–2.26)	< 0.001

*RR* relative risk, *CI* confidence interval, *IQR* inter-quartile range, () %.

**Table 3:** Factors associated with deaths for under-five children hospitalized for pneumonia/severe pneumonia who had their hemoglobin done.

Characteristics	All patients	Death		Unadjusted		Adjusted	
		Yes (n = 194)	No (n = 3274)	OR (95% CI)	p value	OR (95% CI)	p value
Female	1205	90 (46.4%)	1115 (34.1%)	1.68 (1.24–2.27)	< 0.001	1.15 (0.76–1.76)	0.506
Lack of immunization	480	43 (28.9%)	437 (17.3)	1.94 (1.31–2.83)	< 0.001	1.51 (0.94–2.42)	0.091
Abnormal mental status	319	76 (39.6%)	243 (7.6%)	7.94 (5.69–11.01)	< 0.001	2.39 (1.52–3.75)	< 0.001
Severe acute malnutrition	1522	103 (53.1%)	1419 (43.3%)	1.48 (1.10–2.00)	0.008	1.13 (0.73–1.73)	0.591
Dehydration (some/severe)	837	71 (36.5%)	766 (23.4%)	1.89 (1.38–2.58)	< 0.001	0.85 (0.55–1.33)	0.479
Hypoxemia	1083	165 (85.1%)	918 (28.0%)	14.60 (9.71–22.63)	< 0.001	4.28 (2.52–7.27)	< 0.001
Severe sepsis	370	130 (67.0%)	240 (7.3%)	25.68 (18.30–36.16)	< 0.001	7.85 (5.07–12.16)	< 0.001
Anemia	1712	121 (62.4%)	1591 (48.6%)	3.05 (2.71–3.43)	< 0.001	1.88 (1.23–2.89)	0.004
Bacteremia	274	45/172 (26.2%)	229/1644 (13.9%)	2.19 (1.48–3.20)	< 0.001	1.68 (1.01–2.81)	0.047

*OR* odds ratio, *CI* confidence interval, () %.

### Operational definition

Pneumonia/severe pneumonia, severe acute malnutrition, severe underweight, invasive diarrhea, dehydration, hypoxemia, bacteremia, and anemia were defined according to WHO guideline^5^. We have also used evidence-based definition of severe sepsis^14^. Respiratory failure was defined if the proportion of arterial oxygen saturation (measured by using a pulse oximeter) and fraction of inspired oxygen (SpO_2_/FiO_2_) was < 315^15^. Abnormal mental status was defined if a child had restlessness, or lethargy, or coma, or unconsciousness. These variables were shown in Tables 1, 2 and 3.

### Statistical analysis

We analyzed the data using SPSS for Windows (version 20.0) and STATA(version 15.0). For initial baseline analysis (Table 1), anemia was the dependent variable whereas sex, lack of immunization, mental status, severe acute malnutrition, invasive diarrhea, dehydration, hypoxemia, bacteremia, and severe sepsis were the main independent variables. For the outcome table (Table 2) still anemia was the dependent variable whereas respiratory failure, duration of hospital stay and death were the independent variables. The dependent variable of our next analysis (Table 3) was death and main independent variables remained the same in addition to anemia. Normality test was done to understand the distribution of data. We have performed non-parametric test showing median (IQR), if data having continuous variables were not normally distributed. For normally distributed continuous variable we were intending to perform student ‘t’ test showing mean ± SD but we did not have any variable that require this test. We also performed frequencies and percentages for categorical variables. Differences in proportion for categorical variables were compared by the bivariate analysis. Those who had significant associations between the groups (study children who died compared to those who survived) by the bivariate analysis were put into multivariable logistic regression model. In the multivariable logistic regression model death (during hospital course) was the dependent variable and significantly associated factors (on admission) with death that includes anemia were the independent variables. We have also performed Chi-square for linear trend to understand the relation of anemia with increasing age. A p value of less than 0.05 was considered statistically significant. Strength of association was determined by calculating risk ratio (RR) (however, odds ratio [OR] for Table 3) and their 95% confidence intervals (CIs). Additionally, the power of the analysis (anemia vs. non-anemia for outcome of deaths) calculated by using Stata (version 15.0) was 96% with an effect size of 29%.

### Institutional Review Board statement

The name of the ethics committee of icddr,b is “Ethical Review Committee”. All the study protocol was approved by the “Ethical Review Committee” of icddr,b following all methods in accordance with the relevant guidelines and regulations. The approval date of the project was 12 January 2020.

### Informed consent

As it was a retrospective chart review where data were anonymous and no care-giver interview was required, thus, no informed consent was taken. It is important to note that “Ethical Review Committee” of icddr,b waived the need for informed consent of the study.

## Results

### Prevalence and associated factors of anemia

**Figure 1:**
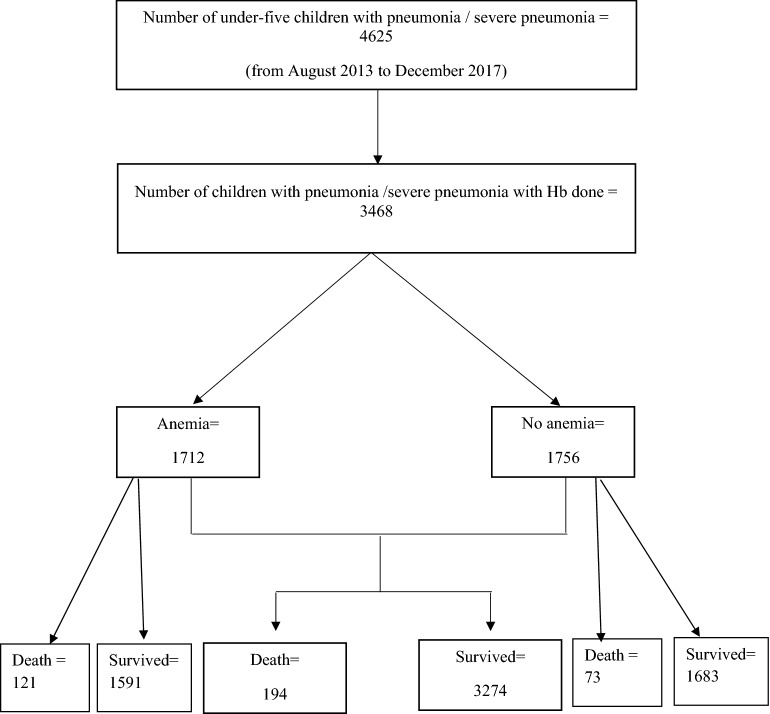
Study profile.

A total of 3468 children fulfilled the inclusion criteria during the study period, and among them,1712 (49.4%) had anemia (Fig. 1). Anemic children more often presented as male (95% CI 1.41–1.51, p < 0.001), had hypoxemia (95% CI 1.09–1.33, p < 0.001), severe acute malnutrition (95% CI 1.01––1.17, p = 0.042), and severe sepsis, compared to those without anemia (Table 1).

### Outcomes of anemic children in the hospital

Anemic children more often developed respiratory failure (95% CI 1.24–2.15, p < 0.001) and fatal outcome (95% CI 1.28–2.26, p < 0.001) than their counterpart (Table 2). A total of 194 (5.6%) children with pneumonia/severe pneumonia had fatal outcome (Fig. 1).

### Factors associated with deaths among study children

Initial bivariate analysis showed that the children with fatal outcome more frequently were female (95% CI 1.24–2.27, p < 0.001), had anemia (95% CI 2.71–3.43, p < 0.001), lack of immunization (95% CI 1.31–2.83, p < 0.001), abnormal mental status (95% CI 5.69–11.01, p < 0.001), severe acute malnutrition (95% CI 1.10–2.00, p = 0.008), dehydration (95% CI 1.38–2.58, p < 0.001), hypoxemia (95% CI 9.71–22.63, p < 0.001), severe sepsis (95% CI 18.30–36.16, p < 0.001), and bacteremia (95% CI 1.48–3.20, p < 0.001) compared with children who survived (Table 3). After adjusting with potential confounders using multivariable logistic regression analysis, we found that anemia (95% CI 1.23–2.89, p = 0.004) remained the independent risk factor for the fatal outcome (Table 3). In the same model, abnormal mental status (95% CI 1.52–3.75, p < 0.001), hypoxemia (95% CI 2.52–7.27, p < 0.001), severe sepsis (95% CI 5.07–12.16, p < 0.001), and bacteremia (95% CI 1.01–2.81, p = 0.047) were also found to be independently associated with fatal outcome (Table 3).

### Relation of anemia with increasing age

**Figure 2:**
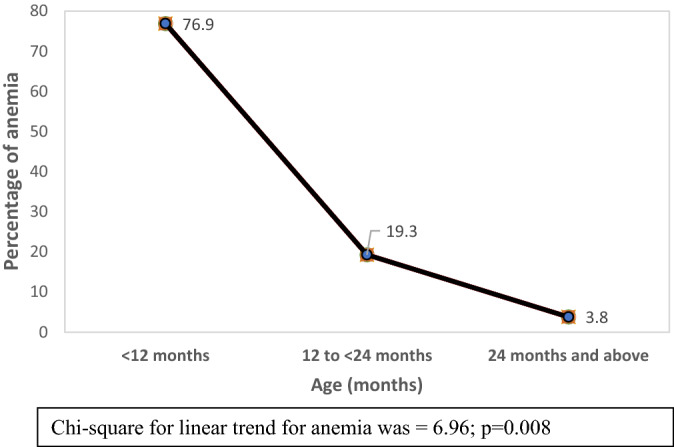
The inverse relation of anemia with increasing age among children under five hospitalized for pneumonia/severe pneumonia using Chi-square for linear trend for anemia.

The trend of prevalence of anemia was found to be inversely related to increasing age among children under five hospitalized for pneumonia/severe pneumonia (Chi-square for linear trend for anemia was = 6.96; p = 0.008) (Fig. 2).

## Discussion

This study identified several important observations: the most important was higher case-fatality rate among the children with anemia compared to those who did not have anemia, and after adjusting for potential confounders anemia remained the independent risk factor for death among the children hospitalized for pneumonia/severe pneumonia. Other important findings includes almost half of the children were anemic, and the trend of anemia was inversely proportional to increasing age among children under five years of age hospitalized for pneumonia/severe pneumonia.

Although, the prevalence of anemia among hospitalized children without specification of pneumonia was found to be variable in a number of previous studies^6–8^, our study is the pioneer, which showed the high prevalence of anemia, specifically in pneumonic children. The prevalence of anemia among children with pneumonia in recent studies from other developing countries was found to be comparatively lower, ranging from 6 to 21.25%^9,16–18^. This observation might be due to the fact that our study children had a number of co-morbidities in addition to pneumonia/severe pneumonia, such as severe acute malnutrition, severe sepsis, and congenital heart disease. In addition, all the studies discussed above used different definitions of anemia, which might impact the variation. It is prudent to mention that the global prevalence of anemia (Hb < 11.0 gm/dl) in this age group of children without specifying the presence of pneumonia is shown to be almost similar (47.4%)^19^ to our study finding. Moreover, data analyzed from Bangladesh government repository found that the prevalence of anemia (without any specification of pneumonia) was 51.9% which is consistent with our study children. Thus, the prevalence of anemia among pre-school children, irrespective of the presence or absence of concomitant pneumonia, is alarmingly high and anemia in this age group demands more attention, especially with co-morbidity, such as with pneumonia/severe pneumonia due to its potentially fatal consequences.

The observation of the trend of prevalence of anemia to be inversely proportional to increasing age among children under five years of age hospitalized for pneumonia/severe pneumonia is really interesting. This might be due to the higher prevalence of severe illnesses, such as severe acute malnutrition^20,21^ and severe sepsis^14^ among infants compared to older children and these severe illnesses were associated with anemia. The observation is almost consistent with a study which evaluated the prevalence of anemia among children from 6 months to 15 years of age with pneumonia in Ecuador^18^.

We found that anemia was associated with abnormal mental status, hypoxemia, and severe sepsis. Simultaneously these three entities were also associated with fatal outcome in our same study population. A number of previous studies identified that abnormal mental status^22^, hypoxemia^14^, and severe sepsis^23^ resemble several pathways leading to fatal outcome of these illnesses. Pneumonic children with hypoxemia used to have a late presentation in the hospital and often suffer from ventilation-perfusion mismatch followed by reduced functional residual capacity of lungs leading to acute respiratory failure and fatal outcome. Children with severe sepsis and/or bacteremia usually present with impaired vaso-regulation as well as reduced cardiac function followed by poor peripheral perfusion^24^, which often leads to fatal outcomes^25^. Any of the above phenomena is found to be featuredwith abnormal mental status of the children^5^.

Thus, our observation of the independent association of anemia with deaths among the children hospitalized for pneumonia/severe pneumonia is explicable. This study had some inherent strengths that included large data set for long duration collected by trained staff. We have also used high quality icddr,b diagnostics for our lab tests. Simultaneously, we had also a number of limitations in this study that included potential selection bias in measuring anemia status, background characteristics were cross sectional in nature, and data collection were mainly done through the routine system.

## Conclusion

Anemia has been shown to be the independent risk factor for deaths among these children during hospitalization. Thus, future studies focusing on anemia during hospitalization of children with pneumonia/severe pneumonia may explore novel interventions that can promote early and appropriate management to reduce morbidity and mortality burden in resource limited settings. The results of our study suggest that the prevalence of anemia was almost 50% and inversely proportional to increasing age among children hospitalized for pneumonia/severe pneumonia. Moreover, prevention of anemia in under-5 children through community-based interventions may reduce the overall burden of childhood pneumonia and severe pneumonia.

## Data Availability

De-identified data will be available upon request from the research administration of icddr, b.
